# Targeted high-throughput sequencing for genetic diagnostics of hemophagocytic lymphohistiocytosis

**DOI:** 10.1186/s13073-015-0244-1

**Published:** 2015-12-18

**Authors:** Bianca Tesi, Kristina Lagerstedt-Robinson, Samuel C. C. Chiang, Eya Ben Bdira, Miguel Abboud, Burcu Belen, Omer Devecioglu, Zehra Fadoo, Allen E. J. Yeoh, Hans Christian Erichsen, Merja Möttönen, Himmet Haluk Akar, Johanna Hästbacka, Zuhre Kaya, Susana Nunes, Turkan Patiroglu, Magnus Sabel, Ebru Tugrul Saribeyoglu, Tor Henrik Tvedt, Ekrem Unal, Sule Unal, Aysegul Unuvar, Marie Meeths, Jan-Inge Henter, Magnus Nordenskjöld, Yenan T. Bryceson

**Affiliations:** Childhood Cancer Research Unit, Department of Women’s and Children’s Health, Karolinska Institutet, Karolinska University Hospital Solna, SE-17176 Stockholm, Sweden; Clinical Genetics Unit, Department of Molecular Medicine and Surgery, and Center for Molecular Medicine, Karolinska Institutet, Karolinska University Hospital, Stockholm, Sweden; Clinical Genetics, Karolinska University Hospital, Stockholm, Sweden; Centre for Infectious Medicine, Department of Medicine, Karolinska Institutet, Karolinska University Hospital Huddinge, SE-14186 Stockholm, Sweden; Department of Pediatrics and Adolescent Medicine, American University of Beirut, Beirut, Lebanon; Department of Pediatric Hematology, Izmir Katip Celebi University Medical Faculty, Tepecik Training and Research Hospital, Izmir, Turkey; Department of Pediatric Hematology Oncology, Istanbul Medical School, Istanbul, Turkey; Department of Oncology and Pediatrics, Aga Khan University, Karachi, Pakistan; Viva-University Children’s Cancer Centre, Department of Paediatric, Yong Loo Lin School of Medicine, National University of Singapore, Singapore, Singapore; Department of Pediatrics, Oslo University Hospital, Oslo, Norway; Department of Pediatrics and Adolescence, PEDEGO Research Unit, Oulu University Hospital, Oulu, Finland; Department of Pediatric Immunology, Erciyes University Medical Faculty, Kayseri, Turkey; Department of Perioperative and Intensive Care, Children’s Hospital, Helsinki University Central Hospital, Helsinki, Finland; Pediatric Hematology Unit of the Department of Pediatrics, Medical School of Gazi University, Ankara, Turkey; Hematology-Oncology Unit, Department of Pediatrics, São João Hospital Center, Oporto, Portugal; Institute of Clinical Sciences, Department of Pediatrics, Sahlgrenska Academy, University of Gothenburg, Gothenburg, Sweden; Queen Silvia Children’s Hospital, Gothenburg, Sweden; Department of Pediatric Hematology and Oncology and Bone Marrow Transplantation Unit, Medipol School of Medicine, Medipol University, Istanbul, Turkey; Department of Medicine, Haukeland University Hospital, Bergen, Norway; Department of Pediatrics, Division of Pediatric Hematology and Oncology, Faculty of Medicine, Erciyes University, Kayseri, Turkey; Department of Pediatrics, Division of Pediatric Hematology, Ankara, Turkey; Division of Pediatric Hematology and Oncology, Istanbul School of Medicine, Istanbul University, Istanbul, Turkey; Broegelmann Research Laboratory, The Gades Institute, University of Bergen, Bergen, Norway

## Abstract

**Background:**

Hemophagocytic lymphohistiocytosis (HLH) is a rapid-onset, potentially fatal hyperinflammatory syndrome. A prompt molecular diagnosis is crucial for appropriate clinical management. Here, we validated and prospectively evaluated a targeted high-throughput sequencing approach for HLH diagnostics.

**Methods:**

A high-throughput sequencing strategy of 12 genes linked to HLH was validated in 13 patients with previously identified HLH-associated mutations and prospectively evaluated in 58 HLH patients. Moreover, 2504 healthy individuals from the 1000 Genomes project were analyzed in silico for variants in the same genes.

**Results:**

Analyses revealed a mutation detection sensitivity of 97.3 %, an average coverage per gene of 98.0 %, and adequate coverage over 98.6 % of sites previously reported as mutated in these genes. In the prospective cohort, we achieved a diagnosis in 22 out of 58 patients (38 %). Genetically undiagnosed HLH patients had a later age at onset and manifested higher frequencies of known secondary HLH triggers. Rare, putatively pathogenic monoallelic variants were identified in nine patients. However, such monoallelic variants were not enriched compared with healthy individuals.

**Conclusions:**

We have established a comprehensive high-throughput platform for genetic screening of patients with HLH. Almost all cases with reduced natural killer cell function received a diagnosis, but the majority of the prospective cases remain genetically unexplained, highlighting genetic heterogeneity and environmental impact within HLH. Moreover, in silico analyses of the genetic variation affecting HLH-related genes in the general population suggest caution with respect to interpreting causality between monoallelic mutations and HLH. A complete understanding of the genetic susceptibility to HLH thus requires further in-depth investigations, including genome sequencing and detailed immunological characterization.

**Electronic supplementary material:**

The online version of this article (doi:10.1186/s13073-015-0244-1) contains supplementary material, which is available to authorized users.

## Background

Hemophagocytic lymphohistiocytosis (HLH) is a severe hyperinflammatory syndrome that presents with unremitting fever, splenomegaly and cytopenia [1]. According to the HLH-2004 protocol, HLH can be defined as fulfillment of at least five of eight clinical and laboratory criteria [2]. Primary, genetic, as well as secondary forms of HLH have been described. HLH is typically treated by immunosuppression, followed by hematopoietic stem cell transplantation in familial cases [1]. Current HLH criteria poorly discriminate underlying causes of disease. Importantly, therapies tailored to different etiologies of HLH may improve treatment outcome [3].

Several genetic disorders predispose to HLH, but vary in their risk of developing disease. Congenital defects affecting the perforin-mediated lymphocyte cytotoxicity, such as autosomal recessive mutations in *PRF1*, *UNC13D*, *STX11*, and *STXBP2*, represent the most common causes of primary HLH, termed familial HLH (FHL) type 2, 3, 4 and 5, respectively [1, 4]. The impaired killing of infected as well as activated immune cells results in the sustained hyperinflammatory state characteristic of HLH, where animal models have postulated a critical role for CD8^+^ T cells and interferon (IFN)-γ [5]. Patients with autosomal recessive mutations in *RAB27A* and *LYST*, causative of Griscelli syndrome type 2 (GS2) and Chediak-Higashi syndrome (CHS), respectively, also frequently develop HLH. Besides defective lymphocyte cytotoxicity, these syndromes are associated with hypopigmentation [6, 7]. Only one case of HLH has been reported in Hermansky-Pudlak syndrome type 2, another hypopigmentation syndrome specifically caused by mutations in *AP3B1* and associated with impaired lymphocyte cytotoxicity [8]. Moreover, HLH has so far not been reported in Hermansky-Pudlak syndrome type 9 patients, caused by mutations in *BLOC1S6* and also reported to display impaired lymphocyte cytotoxicity [9]. Genetic disorders displaying a more limited impairment of lymphocyte cytotoxicity may also present with HLH or related lymphoproliferative diseases. Patients with hemizygous mutations in *SH2D1A* or *XIAP*, associated with X-linked lymphoproliferative disease, typically present with HLH or lymphoproliferative diseases, often triggered by Epstein-Barr virus (EBV) infection [10]. Lymphoproliferation and severe EBV infections are also features of autosomal recessive mutations in *ITK* [11] and hemizygous mutations in *MAGT1* [12], with sporadic cases of HLH [13, 14]. Episodes of HLH have also been reported in patients harboring other primary immunodeficiencies [3, 15–17], providing evidence for hyperinflammatory syndromes fulfilling current HLH criteria in an immunological context of T-cell deficiency or absent IFN-γ signaling. HLH may also arise in the context of inborn errors of metabolism and lysosomal storage disorders, or secondary to infections, malignancies or autoimmune disorders in individuals without any established genetic disease susceptibility [1].

Patients with defective lymphocyte cytotoxicity usually develop early-onset HLH with high penetrance and require the most radical immunosuppressive therapy. Defective natural killer (NK) cell cytotoxic activity, as measured by the ^51^Cr-release assay, is included among the HLH-2004 diagnostic criteria [2]. However, pathological results with this assay do not necessarily reflect functional defects in lymphocyte cytotoxicity, but can also be caused by low NK cell numbers. Refined assays have been developed for the identification of patients with defects in lymphocyte cytotoxicity as well as XIAP signaling [18–20]. These assays require considerable technical expertise and rely on fresh blood samples. Therefore, improved diagnostic procedures are required for guidance of treatment decisions.

With current insights, patients with defective lymphocyte cytotoxicity can also be diagnosed by DNA sequencing. To influence the clinical management of HLH patients, genetic diagnostics must be rapid and accurate. Due to genetic heterogeneity, achieving a molecular diagnosis by conventional Sanger sequencing is labor-intensive and time-consuming. Technological advances have increased sequencing throughput, with decreased sequencing times and costs [21]. As more bench-top sized machines have been pushed to the market, appealing solutions for diagnostic laboratory settings have become available [22]. Aimed at diagnosing a broad range of immune defects, high-throughput assays have recently been reported for the simultaneous study of a number of primary immunodeficiency syndromes [23–25].

Here, we report our experience in implementing a targeted resequencing approach for identification of HLH patients with defective lymphocyte cytotoxicity. Moreover, we characterize the genetic variants in HLH-related genes in the general population and discuss the implications to interpretation of association of rare, potentially damaging monoallelic variants with disease.

## Methods

### Patients

The study was performed using genomic DNA (gDNA) samples from (1) 13 patients with a confirmed molecular diagnosis in an HLH-related gene, (2) 58 patients, prospectively recruited over a 12 months period, fulfilling five or more HLH diagnostic criteria (*n* = 56) or with a cytotoxicity defect suggestive of primary HLH (*n* = 2). Informed consent was obtained from all study participants in accordance with the Declaration of Helsinki. The study was approved by the Regional Ethics Review Board in Stockholm, Sweden.

### AmpliSeq custom panel design

A targeted resequencing panel covering 12 HLH-associated genes was designed according to Ion AmpliSeq technology (Ion Torrent, Thermo Fisher Scientific, MA, USA; Table 1). Using genome build *hg19* as reference, the coding regions, with 25 intronic base pairs around exons, were targeted. For *PRF1*, *UNC13D*, *STX11*, and *STXBP2*, the evolutionarily conserved non-coding regions, identified using the software Alamut (Interactive Bio-software, Rouen, France), were also included. Sequencing data generated by this study has been submitted to the European Genome-phenome Archive and is available from the authors on request.

**Table 1:** Genes included in the panel

Gene	Ensembl gene ID, transcript ID	Locus	Associated disease	Number of amplicons	Coverage (%)	HGMD coverage (%)
*PRF1*	ENSG00000180644, ENST00000373209	10q22.1	Familial hemophagocytic lymphohistiocytosis type 2	11	100	100
*UNC13D*	ENSG00000092929, ENST00000207549	17q25.1	Familial hemophagocytic lymphohistiocytosis type 3	51	98.3	99.2
*STX11*	ENSG00000135604, ENST00000367568	6q24.2	Familial hemophagocytic lymphohistiocytosis type 4	28	100	100
*STXBP2*	ENSG00000076944, ENST00000441779	19p13.2	Familial hemophagocytic lymphohistiocytosis type 5	25	98.5	100
*SH2D1A*	ENSG00000183918, ENST00000371139	Xq25	X-linked lymphoproliferative disease type 1	5	100	98.8
*XIAP*	ENSG00000101966, ENST00000371199	Xq25	X-linked lymphoproliferative disease type 2	13	92.2	92.9
*RAB27A*	ENSG00000069974, ENST00000396307	15q21.3	Griscelli syndrome type 2	10	100	100
*LYST*	ENSG00000143669, ENST00000389794	1q42.3	Chediak-Higashi syndrome	120	97.7	98.4
*AP3B1*	ENSG00000132842, ENST00000255194	5q14.1	Hermansky-Pudlak syndrome type 2	50	94	100
*BLOC1S6*	ENSG00000104164, ENST00000220531	15q21.1	Hermansky-Pudlak syndrome type 9	8	99.1	100
*MAGT1*	ENSG00000102158, ENST00000358075	Xq21.1	X-linked immunodeficiency with magnesium defect, EBV infection, and neoplasia	18	100	100
*ITK*	ENSG00000113263, ENST00000422843	5q33.3	Inducible T-cell kinase deficiency	22	96.7	100

*HGMD* Human Gene Mutation Database

### Library preparation and sequencing

Library preparation was performed with 10 ng gDNA using Ion AmpliSeq Library Kit 2.0 for each multiplex PCR reaction (Ion Torrent, Thermo Fisher Scientific). The library was thereafter ligated with sequencing adaptors containing barcodes (Ion Xpress Barcode Adapters 1–16 Kit, Ion Torrent, Thermo Fisher Scientific). After purification (Agencourt AMPure XP reagent, Beckman Coulter, Brea, CA, USA), libraries were quantified on an Agilent 2100 Bioanalyzer (Agilent Technologies, CA, USA) and diluted to a concentration of 100 pM. Diluted libraries were pooled and further amplified with an emulsion PCR. Enriched templates were loaded onto an Ion 314 or Ion 316 Chip (Ion Torrent, Thermo Fisher Scientific).

### Bioinformatics analyses

Assessment of sequencing quality control, mapping, coverage analysis and variant calling were performed using the Ion Torrent Suite Software (versions 4.0.2 and 4.0.3, Thermo Fisher Scientific). Mapping of sequencing reads to the genome build *hg19* was performed using TMAP software. The Ion Torrent Variant caller (version 4.2.0) was used in “Germline - low stringency” mode with default settings. Integrative Genomics Viewer (IGV) version 2.3.32 [26] was used for the inspection of sequencing reads and visual assessment of detected variants. Called variants were first annotated with Variant Effector Predictor [27] followed by GEMINI (version 0.11.0) [28]. Further analyses were performed with R (version 3.1.2) [29]. In silico evaluation of candidate variants was performed by reviewing CADD [30], PolyPhen-2 [31] and SIFT scores [32]. NNSPLICE 0.9 was used for predicting the effect of splice-site variants [33].

### Sanger validation and sequencing of poorly covered amplicons

All variants considered pathogenic were validated by Sanger sequencing. Moreover, non-covered or poorly amplified exonic regions were Sanger sequenced in unexplained patients. Primers and PCR conditions are available upon request. The PCR products were purified and sequenced on an ABI 3730 Genetic Analyzer (Applied Biosystems, Thermo Fisher Scientific) and analyzed with SeqScape (version 2.5; Applied Biosystem, Thermo Fisher Scientific). Variant segregation in the family was evaluated depending on availability of parental DNA.

### Analysis of mutational load in FHL genes in the 1000 Genomes dataset

Variant call format (vcf) files including sequencing data from 2504 individuals from the 1000 Genomes project were analyzed with respect to genes included in the panel (ftp://ftp-trace.ncbi.nih.gov/1000genomes/ftp/release/20130502/, accessed January 2015). Variants were annotated using Variant Effector Predictor [27] and GEMINI [28]. All variants deviating from Hardy-Weinberg equilibrium (*p* value < 0.05) were removed. Further analyses were performed with R (version 3.1.2).

### Immunological analyses

Intracellular expression of perforin, granzymes, CD107a, and SAP as well as cytotoxic lymphocyte exocytosis was evaluated by flow cytometry [20]. NK cell cytotoxicity against K562 target cells was evaluated with a standard 4-hour ^51^Cr-release assay using peripheral blood mononuclear cells (PBMCs) [34] and the data are shown as lytic units at 25 % specific lysis. All flow cytometry data were acquired on a LSR Fortessa instrument (BD Biosciences, CA, USA). Analyses were performed in Flow Jo v9.7 and R (version 3.1.2).

## Results

### Coverage analysis

**Fig. 1:**
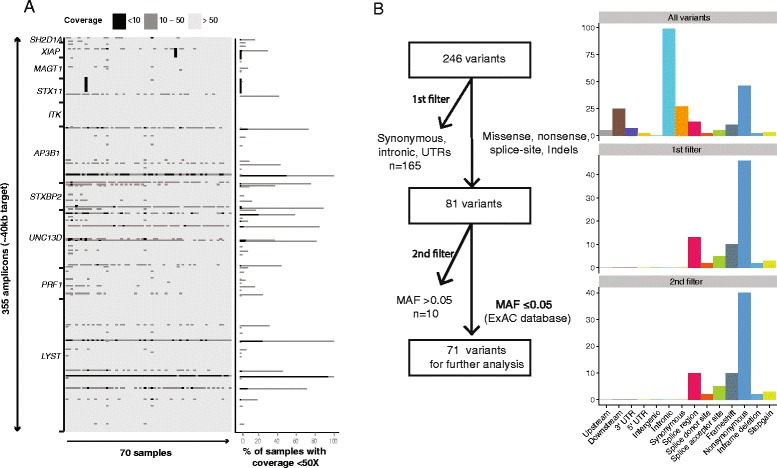
Analysis of coverage efficiency and variant filtering strategy. **a** Heatmap indicating coverage for each individual amplicon (355 amplicons) in each patient sample. The coverage is categorized in <10×, 10–50× or >50× coverage. Patient samples and amplicon are shown in columns and rows, respectively. Patient samples from both the validation and the prospective cohorts are included. Rows are sorted by position. Columns are sorted by average coverage for each patient. On the right side the bar plot is shown the number of samples with lower coverage (<10× and 10–50×). The detailed coverage information of all amplicons is reported in Additional file 1. **b** Flowchart of filtering strategy in the prospective HLH cohort. For each step the bar chart on the right shows the proportions of different types of variants. *MAF* minor allele frequency, *UTR* untranslated region

A custom targeted resequencing panel was designed to cover 12 genes in which mutations have been associated with HLH or lymphoproliferative disorders (Table 1). The panel consisted of 355 primer pairs, with an amplicon size range of 125–175 bp, covering up to 97.3 % of the desired target. Specific primers could not be designed for 1125 bp, due to repetitive regions. Following sequencing, analyses revealed that some amplicons repeatedly failed to generate adequate coverage, as determined by a ≤10× cut-off of mean coverage across samples (Fig. 1a; Additional file 1). Excluding two amplicons that failed in nearly all samples (mean coverage ≤10×), the effective coverage of our initial target sequences was estimated to be 96.6 % of the initial regions of interest, with an average coverage per gene over exonic and splice-site regions of 98 % (Table 1). To ensure clinical efficacy, we calculated the proportion of previously reported mutations (based on the Human Gene Mutation database (HGMD), accessed June 2015) with adequate coverage. Overall, 98.6 % of the mutations listed in HGMD were covered by our design (Table 1).

### Assay validation

To validate our gene panel, we sequenced gDNA from 13 patients with previously identified genetic defects (Table 2). The patients carried a wide spectrum of mutations located in different genes (Table 2). In order to assess the reliability of the method for detection of homozygous exonic deletions, we also included a patient with a 298-bp homozygous exonic deletion of *STXBP2*. We could identify all 18 small genetic aberrations upon read inspection in IGV. Nonetheless, the variant calling software detected only 17 out of 18 small genetic aberrations (Table 2). The *RAB27A* c.148_149delinsC InDel, located in a homopolymer nucleotide stretch, was instead erroneously called as a synonymous variant (c.148A>C; Fig. S1a in Additional file 2). The exonic deletion was easily detected by visual assessment of the coverage over the amplicons (Fig. S1b in Additional file 2).

**Table 2:** Disease-causing mutations used in the validation phase

Gene	Mutation	Effect	Zygosity	Type	Called	Reference
*PRF1*	c.272C>T	p.Ala91Val	Het	Missense	Yes	[52]
*PRF1*	c.797T>C	p.Ile266Thr	Het	Missense	Yes	This study
*UNC13D*	c.2135_2137del	p.Ile712_Gly713delinsSer	Het	Deletion	Yes	This study
*UNC13D*	c.2346_2349del	p.Arg782Serfs*12	Het	Deletion	Yes	[53]
*UNC13D*	c.1388A>C	p.Gln463Pro	Het	Missense	Yes	[41]
*UNC13D*	c.118-307G>A	Reduced expression	Het	Regulatory	Yes	[41]
*UNC13D*	C.2719_2722dup	p.Ser908TyrfsX3	Het	Duplication	Yes	This study
*UNC13D*	c.1992+1G>C	Altered splicing	Het	Splicing	Yes	This study
*STXBP2*	exon 2 deletion	-	Hom	Deletion	Yes^a^	This study
*STXBP2*	c.56T>C	p.Ile19Thr	Het	Missense	Yes	This study
*STXBP2*	c.704G>C	p.Arg235Pro	Het	Missense	Yes	This study
*XIAP*	c.877G>A	p.Gly293Ser	Hemi	Missense	Yes	This study
*XIAP*	c.1141C>T	p.Arg381*	Hemi	Nonsense	Yes	[10]
*RAB27A*	c.148_149delinsC	p.Arg50Glnfs*35	Hom	Indel	No	[54]
*RAB27A*	c.514_518del	p.Gln172Asnfs*2	Hom	Deletion	Yes	[6]
*LYST*	c.2311C>T	p.Gln771*	Hom	Nonsense	Yes	this study
*LYST*	c.1902dup	p.Ala635Serfs*4	Hom	Duplication	Yes	[55]
*AP3B1*	c.1254dup	p.Gln419Thrfs*22	Het	Duplication	Yes	this study
*AP3B1*	c.2626C>T	p.Arg876*	Het	Nonsense	Yes	this study

^a^The *STXBP2* exonic deletion was detected by inspection of coverage plots

*Hemi* hemizygous, *Het* heterozygous, *Hom* homozygous

We next sought to estimate the overall sensitivity of the variant calling strategy by assessing all exonic polymorphisms (*n* = 56) previously identified in the 13 control samples (Additional file 3). In total, 74 variants (*n* = 18 mutations and *n* = 56 polymorphisms) were used for the sensitivity analysis. Out of these, 72 variants were properly called. The overall sensitivity was 97.3 % (95 % confidence interval 90.7–99.2, Wilson score method).

### Prospective cohort of HLH patients

**Fig. 2:**
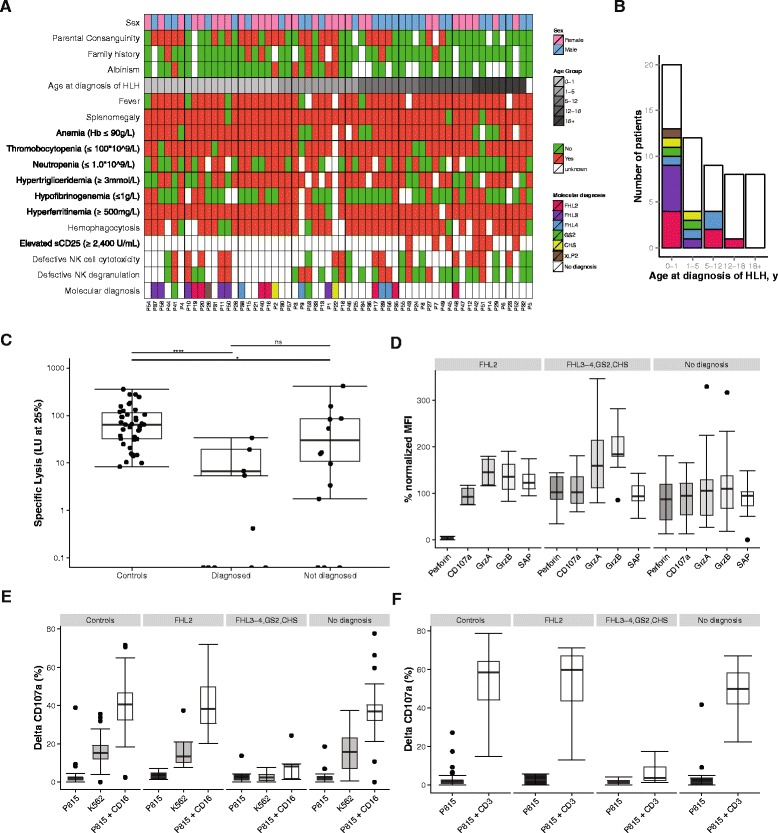
Clinical, genetic and functional characteristics of the patients included in the prospective cohort. **a** Heatmap of clinical and functional features of the implementation cohort in relation to HLH-2004 diagnostic criteria [2]. The patients are ordered based on age at diagnosis of HLH. A family history refers to a positive family history for HLH or unexplained siblings’ death in childhood. **b** The different molecular diagnoses achieved in the prospective cohort according to age group at diagnosis of HLH. **c** NK cell cytotoxic activity, displayed as lytic units at 25 % specific lysis, in healthy controls and patients from the implementation cohort grouped in diagnosed (*n* = 10) and undiagnosed (*n* = 13) (significance level *p < 0.05, ****p < 0.0001). **d** Intracellular expression of perforin, CD107a, granzyme A and B, and SAP in PBMCs of HLH patients from the implementation cohort. Patients are grouped by their molecular diagnosis (*FHL2*, *n* = 4; *FHL3-4,GS2,CHS*, *n* = 9; *No diagnosis*, *n* = 19). The data are expressed as percentage of normalized median fluorescence intensity (*MFI*) in comparison with healthy controls. Exocytic activity of CD3^−^CD56^+^ NK cells (**e**) and CD8^+^CD57^+^ T cells (**f**) was measured as percentage of CD107a^+^ cells in healthy controls and HLH patients from the implementation cohort. P815 target cells were used alone and in combination with anti-CD16 antibody for NK cells and anti-CD3 antibody for CD8 T cells. Exocytic activity of NK cells was also measured using K562 target cells. Patients are grouped by their molecular diagnosis (*FHL2*, *n* = 4; *FHL3-4,GS2,CHS*, *n* = 9; *No diagnosis*, *n* = 20). The controls used were both local and transport controls

Following validation, we sequenced a cohort of 58 prospectively recruited HLH patients (Fig. 2a). The median age at diagnosis of HLH was 3 years, ranging from a few days to 70 years (interquartile range = 0.4–13.2 years; Table 3). Eight patients were above 18 years of age at diagnosis of HLH. The included patients were of different ethnic origin, including 43 % from Turkey. Parental consanguinity was reported in 24 cases. Interestingly, six patients also suffered from albinism and eight patients had a familial history of HLH or unexplained siblings’ death in childhood (Table 3). Hyperferritinemia, splenomegaly and hypertriglyceridemia and/or hypofibrinogenemia were the most common findings in our cohort. Soluble interleukin-2 receptor (sCD25) was elevated in all seven patients tested (Table 3).

**Table 3:** Clinical characteristics of HLH patients included in the prospective cohort

	Whole cohort (%)	Diagnosed (%)	With no diagnosis (%)
Number of patients	58	22	36
Age at diagnosis, years			
0–1	20 of 57 (35)	13 of 22 (59)	7 of 35 (20)
1–5	12 of 57 (21)	4 of 22 (18)	8 of 35 (23)
5–12	9 of 57 (16)	4 of 22 (18)	5 of 35 (14)
12–18	8 of 57 (14)	1 of 22 (5)	7 of 35 (20)
18+	8 of 57 (14)	0 of 22 (0)	8 of 35 (23)
Sex			
Male	28 of 58 (48)	10(45)	18 (50)
Female	30 of 58 (52)	12(55)	18 50)
Parental consanguinity	24 of 52 (46)	14 of 21 (67)	10 of 31 (31)
Familial history of disease	8 of 50 (16)	4 of 8 (50)^a^	4 of 8 (50)^a^
Albinism	6 of 43 (14)	5 of 6 (83)^a^	1 of 6 (17)
Fever	49 of 54 (91)	17 of 20 (85)	32 of 34 (94)
Splenomegaly	55 of 58 (95)	21 of 22 (95)	34 of 36 (94)
Cytopenia (≥2 of 3 lineages)	42 of 48 (88)	15 of 17 (88)	27 of 31 (87)
Anemia	46 of 54 (85)	19 of 21 (90)	27 of 33 (82)
Thrombocytopenia	51 of 57 (89)	19 of 21 (90)	32 of 36 (89)
Neutropenia	26 of 50 (52)	12 of 16 (75)	14 of 34 (41)
Hypertriglyceridemia (≥3 mmol/L)	38 of 51 (75)	13 of 20 (65)	25 of 31 (81)
Hypofibrinogenemia (≤1 g/L)	18 of 50 (36)	7 of 18 (39)	11 of 32 (34)
Hypertriglyceridemia and/or hypofibrinogenemia	46 of 50 (92)	17 of 19 (89)	29 of 31 (94)
Hemophagocytosis	43 of 49 (88)	14 of 17 (82)	29 of 32 (91)
Hyperferritinemia (≥500 mg/L)	54 of 55 (98)	19 of 20 (95)	35 of 35 (100)
Elevated sCD25 (≥2400 U/ml)	7 of 7 (100)	0 of 0	7 of 7 (100)

^a^Shown as the proportion of patients with documented familial disease and albinism, respectively

Overall, 246 genetic variants were identified in the prospective cohort. Filtering for variants with a possible impact at the protein level, and with a minor allele frequency <0.05 in the Exome Aggregation Consortium dataset [35], 71 potentially pathogenic variants were selected for further analysis (Fig. 1b). After manual curation, 19 variants (single-nucleotide variants or small indels), either in homozygous or compound heterozygous state, were classified as disease-causing (Table 4; Additional file 4). One additional disease-causing mutation was found upon read inspection in IGV, namely c.148_149delinsC in *RAB27A* (P53). The same disease-causing mutation was also missed by the variant calling program in our validation study (Table 2; Fig. S1a in Additional file 2).

**Table 4:** Details of disease-causing mutations identified in the prospective cohort

Patient ID	Gene	Mutation	Effect	Zygosity	Type	Associated disease	Reference
P48	*PRF1*	c.272C>T	p.Ala91Val	Het	Missense	FHL2	[52]
		c.1288G>T	p.Asp430Tyr	Het	Missense		[56]
P20	*PRF1*	c.659G>A	p.Gly220Asp	Hom	Missense	FHL2	This study
P35	*PRF1*	c.673C>T	p.Arg225Trp	Hom	Missense	FHL2	[57]
P16, P40	*PRF1*	c.1122G>A	p.Trp374*	Hom	Nonsense	FHL2	[57]
P17	*PRF1*	c.1349C>T	p.Thr450Met	Hom	Missense	FHL2	[58]
P19	*PRF1*	c.1179C>A	p.Cys393*	Het	Nonsense	FHL2	This study
		c.1434G>T	p.Leu478Arg	Het	Missense		This study
P11	*UNC13D*	c.569+5G>A	Altered splicing^b^	Het	Splicing	FHL3	[53]
		inversion^a^		Het	Inversion		[34]
P1	*UNC13D*	c.570-1G>A	Altered splicing^b^	Hom	Splicing	FHL3	This study
P37	*UNC13D*	c.753+1G>T	Altered splicing^b^	Hom	Splicing	FHL3	[59]
P10	*UN13D*	c.2236C>T	p.Gln746*	Het	Nonsense	FHL3	This study
		c.2346_2349del	p.Arg748Serfs*12	Het	Deletion		
P50	*UNC13D*	c.2709+2T>A	Altered splicing^b^	Hom	Splicing	FHL3	This study
P58	*UNC13D*	c.2544delT	p.Ile848Metfs*67	Hom	Deletion	FHL3	This study
P9, P38, P39	*STX11*	c.369_376delinsTGG	p.Val124Glyfs*60	Hom	Indel	FHL4	[60]
P56	*STX11*	Exonic deletion	-	Hom	Large deletion	FHL4	NA
P26	*XIAP*	Exonic deletion	-	Hemi	Large deletion	XLP2	NA
P2	*LYST*	c.9107-20_9109_del	Altered splicing	Hom	Splicing	CHS	[61]
P22	*LYST*	c.2749_2750del	p.Arg917Glyfs*5	Hom	Deletion	CHS	This study
P53	*RAB27A*	c.148_149delinsC	p.Arg50Glnfs*35	Hom	Indel	GS2	[54]
P41	*RAB27A*	c.514_518del	p.Gln172Asnfs*2	Hom	Deletion	GS2	[6]

^a^ The *UNC13D* inversion was detected with a specific multiplex PCR assay [34]

^b^ As predicted by NNSPLICE 0.9

*Hemi* hemizygous, *Het* heterozygous, *Hom* homozygous, *NA* not applicable, *XLP* X-linked lymphoproliferative disease type 2

In addition to analysis of single nucleotide variants and small indels, we performed coverage analysis to identify larger homozygous deletions (Fig. 1a). We identified a hemizygous deletion of *XIAP* in P26 and a large homozygous deletion of *STX11* in P56 (Fig. S1c, d in Additional file 2).

### Molecular diagnoses

In total, we identified and validated 22 unique disease-causing mutations located in six different genes (Table 4), achieving a molecular diagnosis in 22 patients (overall diagnostic rate 38 %, 22 of 58). The diagnostic yield was higher, 65 % (13 of 20), in the group of patients with HLH presentation before one year of age compared with 24 % (9 of 37) among the older patients (Fig. 2b). Excluding adult cases of HLH (*n* = 8), the diagnostic rate was 44 % (22 of 50) among the pediatric cases. Interestingly, the oldest patient with primary HLH in this cohort, aged 16 years (P48), had compound heterozygous variants in *PRF1*, c.272C>T (p.Ala91Val) and c.1288G>T (p.Asp430Tyr). The variant p.Ala91Val has been associated with later onset of disease when in *trans* to other *PRF1* mutations [36].

We identified patients with biallelic mutations in *PRF1* (*n* = 7), *UNC13D* (*n* = 6), *STX11* (*n* = 4), *RAB27A* (*n* = 2) and *LYST* (*n* = 2) as well as a patient with a hemizygous mutation in *XIAP* (Fig. 2b, Table 4). The mutational spectrum was broad, including missense, nonsense, and splicing mutations, indels, small and large deletions. Eight of the 22 identified mutations are novel (Table 4). Mutations were identified in five out of six patients presenting with albinism and HLH. Interestingly, one such patient with albinism (P1) was diagnosed with a homozygous *UNC13D* splice-site mutation (c.570-1G>A). No patients were found to have mutations in *STXBP2*, *SH2D1A*, *ITK*, *MAGT1*, *AP3B1*, and *BLOC1S6*.

Finally, in 36 out of 58 patients, biallelic variants that could explain the disease phenotype were not detected. The age at diagnosis of HLH was significantly higher compared with genetically diagnosed patients (Wilcoxon rank sum test, *p* = 0.06). Moreover, 61 % (22 of 36) were diagnosed with a concomitant disease known to predispose to secondary HLH. The most commonly associated diseases were EBV infection (*n* = 10), other infections (*n* = 4), and hematological cancer (*n* = 3). In contrast, an infectious trigger was reported in only 4 of 22 (18 %) of the genetically diagnosed patients. Thus, the group of undiagnosed patients had a higher frequency of known triggers of HLH (Fisher’s exact test, *p* = 0.002). Of note, seven out of the eight adult HLH cases (88 %) were associated with a known trigger of HLH, suggesting that these may truly represent secondary HLH cases.

### Correlation between genetic and functional findings

Results from at least one NK cell or CD8^+^ T cell functional assay were available from 33 patients, including 13 patients with a molecular diagnosis and 20 patients without a definitive diagnosis. Substantiating the genetic findings, immunological analyses revealed defective NK cell cytotoxicity and virtually absent perforin expression in the four patients with biallelic *PRF1* mutations studied (Fig. 2a, c, d). Moreover, CD8^+^ T-cell and NK cell exocytosis was defective in patients with mutations in *UNC13D*, *STX11*, *RAB27A*, and *LYST* (Fig. 2a, e, f). Interestingly, a patient with *RAB27A* mutations presented with defective NK and CD8^+^ T cell exocytosis, but normal NK cell cytotoxicity, while a patient with *LYST* mutations displayed defective NK cell cytotoxicity but only abnormal NK and CD8^+^ T-cell exocytosis (Fig. 2a, d, e). Overall, all patients with a genetic diagnosis for which functional data were available displayed a functional defect by at least one diagnostic assay.

Among the undiagnosed patients, seven patients displayed defective NK cell cytotoxicity (<10 LU; *n* = 6) and/or exocytosis (<5 % CD107a^+^ NK cells following K562 target cell incubation; *n* = 4). Three such cases belonged to the adult HLH cohort (Fig. 2a). Therefore, 4 out of 15 pediatric HLH cases (26 %) for which functional data were available displayed a defective NK cell function. In a few cases, the low NK cell cytotoxicity could reflect the low percentage of NK cells in PBMCs (Additional file 5). Notably, none of the undiagnosed patients with defective exocytosis against K562 target cells displayed concomitant defective NK cell exocytosis following anti-CD16 stimulation or defective CD8^+^CD57^+^ T-cell exocytosis following anti-CD3 stimulation (Fig. 2e). This result contrasted patients with biallelic *UNC13D*, *STX11*, *STXBP2*, *RAB27A*, or *LYST* mutations, which all displayed defective exocytosis in response to all stimuli. A greater variability has been noted in assays quantifying NK cell exocytosis in response to Fc receptor engagement or CD8^+^CD57^+^ T-cell exocytosis in response to T-cell receptor engagement [20]. Taken together, it is possible that impaired K562 target cell-induced exocytosis in these patients does not reflect mutations in proteins generally required for cytotoxic lymphocyte exocytosis.

### Contribution of monoallelic mutations as cause of HLH

In patients without an established molecular diagnosis based on biallelic or hemizygous mutations, we identified seven different monoallelic variants in nine patients with damaging predictions by either SIFT or PolyPhen-2 (Additional file 6). These were regarded as monoallelic variants of unknown significance. Three patients carried the variant *PRF1* c.272C>T p.Ala91Val in a heterozygous state. One of these patients also carried an additional rare variant with pathogenic prediction in *STXBP2* (c.1034C>T, p.Thr345Met), a combination previously reported in two patients with HLH [37]. The monoallelic variants were identified among both pediatric (*n* = 7) and adult (*n* = 2) patients. Four out of the nine patients with monoallelic variants were reported to have a known trigger of HLH and only one had a positive family history of unexplained siblings’ death in childhood. Overall, 25 % of HLH patients without an established molecular diagnosis carried at least one variant with an in silico damaging prediction.

To interpret findings in patients without an established molecular diagnosis and provide an overview of genetic variations in genes linked to HLH, we examined the frequency of variants in genes included in our panel among 2504 unrelated individuals from the 1000 Genomes project. Discarding intronic variants outside splice-site regions and synonymous variants, 1956 individuals carried at least one variant with a minor allele frequency lower than 0.05. Applying more strict filters (i.e., at least one damaging prediction by either SIFT or PolyPhen-2), 636 individuals (25.4 %) were identified as carrying at least one possibly damaging variant (Additional file 7). The majority of variants were found in *LYST* and *UNC13D*, likely reflecting gene size (Fig. S3a, b in Additional file 8). Limiting the analysis to FHL genes, 413 individuals carried at least one possibly damaging variant. Surprisingly, monoallelic variants in genes linked to HLH were thus not enriched in patients with HLH lacking a molecular diagnosis (Fig. S3c, d in Additional file 8). Nonetheless, the *PRF1* c.272C>T (p.Ala91Val) variant, in a heterozygous state, showed a weak enrichment (Fisher’s exact test, *p* value = 0.07) in patients with HLH but no biallelic mutations. However, a larger cohort of HLH patients lacking biallelic mutations is required to study this association further. Of note, two individuals out of 2504 were homozygous for possibly damaging variants in HLH-related genes, namely *PRF1* c.272C>T (p.Ala91Val) and *UNC13D* c.1579C>T (p.Arg527Trp).

## Discussion

Current HLH diagnostic criteria are not specific in discriminating patients with a strong genetic predisposition, typically involving defects in lymphocyte cytotoxicity, from those with a number of other etiologies also associated with HLH [1, 3]. As treatment recommendations may differ between the groups, molecular studies can have a direct impact on clinical management. Recent technological advances in DNA sequencing have enabled more comprehensive approaches for genetic screening [38–40]. In this study, we developed and validated a targeted high-throughput sequencing approach in order to rapidly identify patients with mutations in genes required for lymphocyte cytotoxicity. Furthermore, we tested the efficacy of our approach on a prospective cohort of 58 patients fulfilling clinical HLH criteria.

Twelve genes, previously linked to HLH, albinism with HLH, or susceptibility to severe EBV infections, were included in our panel. Overall, we achieved 96.6 % coverage of the genes of interest, with an average coverage per gene of 98 %. Moreover, our design covered almost all sites previously reported as mutated. Lack of coverage was mainly due to difficulty of primer design in repetitive regions. Of the designed primer pairs, only a few amplicons failed sequencing. While panels for the simultaneous analysis of several primary immunodeficiency genes have recently been described [23–25], this is the first report of an HLH gene-specific panel implemented on a substantial number of HLH patients. To our knowledge, this also represents the first resequencing panel that targets evolutionarily conserved intronic regions, which is of importance because such regions harbor disease-causing mutations in HLH patients [34, 41]. The combination of exonic and intronic targets and the spectrum of genes targeted make our panel a comprehensive solution for the molecular diagnostics of HLH patients.

For validation of our variant calling strategy we analyzed 13 patients with a known molecular diagnosis that harbored 18 different mutations and 56 additional variants. The patients were selected in order to cover different kinds of mutations distributed over multiple genes. Our analysis revealed a sensitivity of 97.3 %, comparable to other panels based on Ion Torrent technology [24]. The only missed disease-causing mutation was located in a homopolymer stretch, regions that are challenging to sequence with Ion Torrent technology [42–44]. All variants were correctly visualized in IGV, suggesting that sensitivity potentially can approximate 100 % through further optimization of variant calling software.

When applied to a heterogeneous cohort of 58 patients, with a clinical diagnosis of HLH (*n* = 56) or with a functional defect suggestive of primary HLH (*n* = 2; defective NK cell activity combined with defective exocytosis or decreased perforin expression), we identified 22 disease-causing mutations, of which eight were novel, in six of the twelve genes included in the panel. In agreement with the results from the validation cohort, the spectrum of mutations identified in the prospective cohort clearly demonstrated that our method identified different kinds of mutations, including a 22-bp homozygous deletion in *LYST*. Notably, three patients were found to carry a homozygous indel (*STX11* c.369_376delinsTGG), which was not detected in another resequencing study [23]. Moreover, large homozygous deletions were readily identified by coverage analysis and inspection of sequencing reads in both the validation and implementation cohorts. Overall, we achieved a definitive molecular diagnosis in 22 patients (38 %). Biallelic mutations in *PRF1* (*n* = 7) and *UNC13D* (*n* = 6) represented the most common finding.

The diagnostic yield was high in the group of patients diagnosed with HLH before one year of age (65 %). In the largest collection of patients with a suspected diagnosis of HLH studied for mutations in *PRF1*, *UNC13D* and *STXBP2*, biallelic mutations were found in 11 % of all cases, and 24 % of cases with onset of disease before 1 year of age [45]. The larger proportion of patients with a genetic diagnosis in our cohort may reflect the larger number of genes studied, more specific inclusion criteria, and a high frequency of consanguinity. Rather, our results compare well with data from the Italian HLH registry, where 40 % of HLH patients overall received a definitive molecular diagnosis [46]. In this cohort, 64 % of HLH patients with an age at onset below 1 year received a molecular diagnosis. Of note, one patient with albinism and HLH was reclassified post-sequencing as FHL3, illustrating a case where phenotypic characteristics potentially could misguide targeted genetic investigations. Conversely, genetic analyses can correct for overlooked phenotypic manifestations [47]. Moreover, *RAB27A* mutations have recently also been reported in patients without albinism, necessitating *RAB27A* sequencing in all HLH patients with defective exocytosis [48].

Despite our efforts, 36 patients remained without a definitive molecular diagnosis. Bioinformatics analyses were complemented by visual inspection of sequencing reads and Sanger sequencing of poorly covered amplicons, reducing the likelihood of overlooking mutations. Regulatory mutations or mutations in genes not included in this panel, as well as secondary forms of HLH, are plausible explanations for the lack of genetic findings in these patients. For instance, our small cohort of adult HLH cases may represent secondary HLH. Nonetheless, adult HLH cases should still be studied for primary HLH, as a relatively small proportion of them do harbor biallelic mutations in HLH-related genes [45, 46, 49]. For three undiagnosed patients with a family history of unexplained siblings’ death in childhood, the medical records of the siblings’ disease were scarce. Thus, we could not be sure that these represented familial HLH. Conversely, the sister of an additional undiagnosed patient with EBV-driven HLH (P7) also suffered from a prolonged EBV infection with hepatitis, leukopenia, anemia, and prolonged fever at the age of 7 years, suggesting a familial susceptibility to severe EBV infections in this family. Generally, undiagnosed patients had a higher median age at diagnosis and frequency of known triggers for secondary HLH, with the most common trigger being EBV infection. Four undiagnosed pediatric HLH patients displayed defective NK cell cytotoxicity and/or a selective defect in NK cell exocytosis against K562 target cells, possibly suggesting an immune defect more restricted to NK cell function in these patients.

Interestingly, rare monoallelic variants in genes required for lymphocyte cytotoxicity have previously been reported in HLH patients [37, 45]. However, their contribution to disease development is unclear. We identified nine patients with seven distinct rare monoallelic variants with in silico pathogenic predictions, without any obvious enrichment by age at onset or HLH trigger. Three patients carried the variant *PRF1* p.Ala91Val. To gain understanding of the role of monoallelic variants in HLH, we determined the mutational load of the genes included in our panel among 2504 adults from the 1000 Genomes project [50]. Remarkably, in comparison to our cohort of undiagnosed patients and even to a larger cohort from the Italian registry with 18 % of monoallelic variants in sporadic HLH [46], a similar frequency of rare possibly pathogenic variants was found in the 1000 Genomes cohort. Of the 25 rare, possibly pathogenic heterozygous *PRF1* variants identified in the 1000 Genomes project cohort, 11 (36 %) have previously been reported as homozygous or compound heterozygous mutations in patients diagnosed with FHL2. Although rare genetic variants may contribute to disease susceptibility, such conclusions require more rigorous experimental validations as recently exemplified for a dominant-negative *STXBP2* variant [51]. Although limited in scale and demography, our results suggest a similar burden of heterozygous variants with pathogenic prediction in HLH-associated genes between HLH patients without a known genetic defect and healthy individuals. Thus, prudence is warranted with respect to interpreting causality between rare monoallelic variants and HLH.

## Conclusions

We have demonstrated the efficacy of a high-throughput sequencing approach for the molecular diagnosis of patients with suspected HLH. With more than half of the patients lacking an identified genetic aberration, the genetic susceptibility to HLH remains to be discovered with further genome sequencing and immunological characterization. Moreover, we determined the burden of heterozygous variants with pathogenic prediction of HLH-related genes in the general population and unexpectedly found it similar to that observed in HLH patients without a clear genetic diagnosis. Albeit based on a small cohort, our results imply prudence in ascertaining any causality between monoallelic mutations and HLH. Despite the good accuracy in high-throughput sequencing, such diagnostic approaches are best combined with sensitive functional assays for reliable molecular diagnoses of patients with HLH.

## Additional files

Additional file 1: Table S1. Coverage information for each amplicon. (XLSX 73 kb) Additional file 2: Figure S1. (**a**) IGV screenshot over the *RAB27A c.148_149delinsC* indel missed by the variant calling. (**b**) Coverage distribution over the *STXBP2* gene in a control patient and a patient with a deletion of exon 2. (**c**) Coverage distribution over the *STX11* gene in a control patient and in P56, a patient with a *STX11* deletion. (**d**) Coverage distribution over the *XIAP* gene in a control patient and in P26, a patient with a *XIAP* deletion. (EPS 3130 kb) Additional file 3: Table S2. Polymorphisms and rare variants used in the validation analysis. (DOCX 19 kb) Additional file 4: Table S3. Classification of rare variants identified in the prospective cohort. (DOCX 56 kb) Additional file 5: Figure S2. Scatterplot of NK cell cytotoxic activity, displayed as lytic units at 25 % specific lysis, and frequency of NK cells in PBMCs. The patients are grouped by their molecular diagnosis. The individuals with very low NK cytotoxicity are also displayed in the zoomed plot for better resolution. Transport controls are included (*triangles*). (EPS 1171 kb) Additional file 6: Table S4. Monoallelic rare variants with in silico pathogenic predictions identified in the patients without a definitive diagnosis. (XLSX 38 kb) Additional file 7: Table S5. Monoallelic rare variants with in silico pathogenic predictions identified in the 1000 Genomes cohort. (XLSX 100 kb) Additional file 8: Figure S3. Distribution of all variants (**a**) and variants with likely pathogenic effect (**b**) by gene. Frequency of at least one rare variant with pathogenic in silico prediction in the 1000 Genomes cohort versus the undiagnosed patients in all 12 genes analyzed (**c**) or in the FHL genes only (**d**). (EPS 1784 kb)

## Abbreviations

CHS: Chediak-Higashi syndrome
EBV: Epstein-Barr virus
FHL: familial hemophagocytic lymphohistiocytosis
gDNA: genomic DNA
GS2: Griscelli syndrome type 2
HGMD: Human Gene Mutation Database
HLH: hemophagocytic lymphohistiocytosis
IFN: interferon
IGV: Integrative Genomics Viewer
NK: natural killer
PBMC: peripheral blood mononuclear cells
