# Supplementary material for: Targeted high-throughput sequencing for genetic diagnostics of hemophagocytic lymphohistiocytosis
Source: Genome Med. 2015 Dec 18;7:130. doi: 10.1186/s13073-015-0244-1 (PMC4684627; doi:10.1186/s13073-015-0244-1)
Supplement: Additional file 3: Table S2. — Polymorphisms and rare variants used in the validation analysis. (DOCX 19 kb) [file 13073_2015_244_MOESM3_ESM.docx]

| Gene | Variant | rs ID | MAF | Called |
| --- | --- | --- | --- | --- |
| *PRF1* | c.272C>T | rs35947132 | 0.02 | 1/1 |
| *PRF1* | c.822C>T | rs885821 | 0.13 | 1/1 |
| *PRF1* | c.900C>T | rs885822 | 0.31 | 2/2 |
| *UNC13D* | c.175G>A | rs9904366 | 0.01 | 2/2 |
| *UNC13D* | c.279C>T | rs3744007 | 0.12 | 1/2 |
| *UNC13D* | c.811C>T | rs139564938 | - | 1/1 |
| *UNC13D* | c.888G>C | rs7223416 | 0.49 | 8/8 |
| *UNC13D* | c.1723G>C | - | - | 1/1 |
| *UNC13D* | c.1744C>T | rs75853379 | 0.03 | 1/1 |
| *UNC13D* | c.2599A>G | rs1135688 | 0.49 | 6/6 |
| *UNC13D* | c.2782C>T | rs35037984 | 0.01 | 1/1 |
| *UNC13D* | c.3198A>G | rs7210574 | 0.48 | 7/7 |
| *STXBP2* | c.165C>T | rs11538945 | 0.007 | 1/1 |
| *STXBP2* | c.849G>A | rs34450592 | 0.01 | 1/1 |
| *STXBP2* | c.1443C>T | rs10001 | 0.48 | 6/6 |
| *STXBP2* | c.1576A>G | rs6791 | 0.26 | 8/8 |
| *STXBP2* | c.1663A>G | rs61736586 | 0.01 | 1/1 |
| *XIAP* | c.1268A>C | rs5956583 | 0.33 | 2/2 |
| *AP3B1* | c.1683C>T | rs17192146 | 0.04 | 1/1 |
| *AP3B1* | c.1754T>A | rs6453373 | 0.18 | 2/2 |
| *BLOC1S6* | c.144A>T | - | - | 1/1 |

**Table S2.** Polymorphisms included in the validation analysis.
