# Supplementary material for: Targeted high-throughput sequencing for genetic diagnostics of hemophagocytic lymphohistiocytosis
Source: Genome Med. 2015 Dec 18;7:130. doi: 10.1186/s13073-015-0244-1 (PMC4684627; doi:10.1186/s13073-015-0244-1)
Supplement: Additional file 4: Table S3. — Classification of rare variants identified in the prospective cohort. (DOCX 56 kb) [file 13073_2015_244_MOESM4_ESM.docx]

| Classification | # of variants |
| --- | --- |
| Disease-causing | 19 |
| Benign based on previous reports | 3 |
| Biallelic VUS | 1 |
| Monoallelic VUS | 13 |
| Heterozygous in XLR genes (females) | 3 |
| Not fitting with phenotype | 21 |
| Found in patients with an alternative diagnosis | 6 |
| False positives | 4 |
| Annotation error | 2 |
| Total | 72* |

**Table S3.** Classification of rare variants identified in the implementation cohort

*A91V is counted twice, both as disease-causing and monoallelic VUS; VUS = variants of unknown significance
